# Vagus nerve stimulation for treatment-resistant mood disorders: a long-term naturalistic study

**DOI:** 10.1186/s12888-015-0435-8

**Published:** 2015-03-31

**Authors:** Umberto Albert, Giuseppe Maina, Andrea Aguglia, Alberto Vitalucci, Filippo Bogetto, Chiara Fronda, Alessandro Ducati, Michele Lanotte

**Affiliations:** 1 Rita Levi Montalcini Department of Neuroscience, Mood and Anxiety Disorders Unit, University of Torino, via Cherasco 11, 10126 Turin, Italy; 2 Rita Levi Montalcini Department of Neuroscience, Neurosurgery Unit, University of Torino, via Cherasco 11, 10126 Turin, Italy

**Keywords:** Treatment resistant depression, Vagus nerve stimulation, Long-term follow-up, Neuromodulation

## Abstract

**Background:**

Limited therapeutic options are available for patients with treatment-refractory major depression who do not respond to routinely available therapies. Vagus nerve stimulation showed adjunctive antidepressant effect in chronic treatment resistant depression, even though available studies rarely exceed 2-year follow up.

We report a naturalistic 5-year follow up of five patients who received VNS implant for resistant depression (3 patients with major depressive disorder and 2 with bipolar disorder).

**Methods:**

Response was defined as a reduction of the 17-item HDRS total score ≥50% with respect to baseline, remission as a score ≤7.

**Results:**

Response and remission rates were both 40% (2/5) after 1 year, and 60% (3/5) at 5 years. Two patients withdrew from the study because of side effects or inefficacy of stimulation.

**Conclusions:**

Our case series showed that long-term VNS may be effective in reducing severity of depression in a small but significant minority of patients, although two patients had stimulation terminated because of adverse effects and/or refusal to continue the study.

## Background

Major Depressive Episode (MDE) is a clinical condition affecting about 350 million people worldwide, carries a high morbidity and mortality and imposes significant costs on patients, their families, caregivers, employers, and insurance payers [1].

Despite considerable efforts made over the past decades, there are a large proportion of patients who still do not respond to currently available treatments [2,3]. Up to 50% of subjects with a MDE who receive an antidepressant treatment do not respond satisfactorily to the first trial and another 50% of them do not fully respond to a second antidepressant medication [4,5]. Furthermore, patients often show relapses despite treatment with conventional medication [6].

There is not a general consensus on how many unsuccessful trials are required to meet criteria for treatment-resistant depression (TRD), and there are several ways of staging this condition [7-10]. Despite the lack of a unique definition, TRD can be defined as the absence of response to at least two antidepressant trials given in succession at adequate doses and duration in compliant subjects [2]. Rates of TRD range from 2-3% [4] to 30% [11] and vary according to the threshold of unsuccessful trials. Treatment-resistant depression (TRD) remains however a significant clinical challenge, irrespective of the definition used.

Vagus Nerve Stimulation (VNS) is currently approved both in Europe and in the USA as an adjunctive long-term treatment of chronic (actual episode ≥2 years) or recurrent depression for patients aged 18 years or older who are experiencing a MDE (both unipolar and bipolar) and have not had a sufficient response to four or more adequate antidepressant treatments [12].

VNS therapy® (Cyberonics, Inc, Houston, TX, USA) consists in implanting a generator in the left chest and connecting it to the left vagus nerve with a bipolar lead [13]. After generally 2 weeks from surgery, a wand connected to a hand-held computer activates the device telemetrically. The device provides intermittent stimulation to the left vagus nerve; the electrical signals are in turn processed in the nucleus tractus solitarius and relayed to various regions of the brain to provide relief of depressive symptoms through mechanisms not yet fully understood [14].

In recent years, several studies have been performed examining VNS efficacy in MDEs, and reviews are already available on several clinical and experimental issues concerning VNS in TRD [15,16]. VNS appears to be effective acutely in a small but significant proportion of patients (15.2-57%); moreover, response rates increase from 3 months to 1 or 2 years [5,17-34].

The time course for the clinical response to VNS suggests that when clinical improvement occurs, it requires weeks or months to become evident. As such, VNS may prove more useful as a long-term maintenance therapy for chronic depression rather than for acute stabilization of an episode.

The effectiveness of long-term VNS in clinical practice remains, however, to be determined; the question of whether VNS is helpful to non-research, treatment-seeking patients is still open. Moreover, there is a lack of knowledge regarding long-term follow-up of patients who underwent VNS therapy: available data do not exceed 2 years, except a case report of a 38-year old woman with a six-year follow-up [35] and a case series recently published with 7 patients who were re-evaluated 48–60 months after surgery [36].

The aim of the present paper is to present data of a naturalistic 5-year follow-up of patients who received VNS as an adjunctive treatment for resistant depression.

## Methods

### Participants

Participants were recruited consecutively from January 2007 to May 2008 among subjects referred to the Mood and Anxiety Disorders Unit of the Department of Neuroscience, University of Turin (Italy); this is a tertiary referral centre located within the University Hospital and specialized in the treatment of patients with Mood Disorders.

The aims of the study as well as study procedures were thoroughly explained to potential participants who gave written consent before participation. The study design was reviewed and approved by the local ethics committee (Comitato Etico Interaziendale A.O.U. Città della Salute e della Scienza di Torino - A.O. Ordine Mauriziano - A.S.L. TO1).

To be enrolled in the study, patients fulfilled the following inclusion criteria: a) a current Major Depressive Episode, chronic (actual episode ≥2 years) or recurrent (history of at least 4 lifetime MDEs), according to the Structured Clinical Interview for Axis I Disorders (SCID-I/P – DSM IV TR) [37]; b) 18 years of age or older; c) during the current MDE, failure to respond to at least two adequate trials of antidepressant treatments; d) a minimum total score of 20 on the HDRS; and e) stable psychopharmacological medication for at least 4 weeks before baseline. We defined non-responder a patient who failed to show a reduction of the 17-item HDRS total score ≥50% with respect to the beginning of the pharmacological trial. We considered an adequate antidepressant trial, according to Sackeim [2], a full-dosage antidepressant given for at least 4 weeks. We also required that patients took two antidepressants of different pharmacological classes.

Exclusion criteria were: 1) past or current presence of psychotic features; 2) suicide attempt requiring medical treatment within the previous twelve months; 3) history of schizophrenia, schizoaffective disorder or rapid cycling bipolar disorder; 4) severe Axis II disorders (such as borderline or antisocial); 5) alcohol or substance dependence within the previous twelve months or abuse of a substance other than nicotine during the previous six months; 6) diagnosis or signs of delirium, dementia, or amnestic and other cognitive disorders; 7) previous head injuries, cardiac or neurological diseases, and surgical implantation-related risks.

All patients were on antidepressants (with or without mood-stabilizers depending on the diagnosis of MDD or BD), which were not withdrawn after surgery. Throughout the follow-up period, the dosage of antidepressant medications was adjusted according to clinical condition, while no change of type of antidepressant treatment or mood-stabilizer was performed during the first year after surgery.

### Clinical assessment

Unmasked clinical outcome measures included the 17-item Hamilton Rating Scale for Depression (HDRS-17), the Montgomery-Asberg Depression Rating Scale (MADRS), and the Clinical Global Impressions-Severity of Illness (CGI-S) scale. These measurements were obtained at pre-treatment (baseline), at post-surgery (2 weeks after implantation), every three months for the first two years and then annually. During the first 12 months, a self-report measure of quality of life was also administered: the Medical Outcomes Study 36-Item Short-Form Health Survey (SF-36). The SF-36 contains 8 scales for assessing physical functioning, role limitations due to physical health, bodily pain, general health, vitality, social functioning, role limitations due to emotional problems, and mental health. Summary scales include a physical composite and a mental composite that are expressed as t scores (mean = 50, SD = 10).

Response was defined as a reduction of the 17-item HDRS total score ≥50% with respect to baseline, remission as a score ≤7.

In addition, tolerability and side effects were recorded. Adverse events were defined as events occurring on or after the date of implantation, events not reported as signs or symptoms at baseline and/or worsening in severity or frequency. Presence of mania was monitored using the Young Mania Rating Scale (YMRS): a score of 12 was used as the threshold for the diagnosis of mania.

### Operative procedure

The device component consists of a pulse generator with a lithium battery and a lead wire with two helical electrodes and a tethering anchor. Under operating microscope the helical electrodes and the anchor tether coil were wrapped around the left cervical vagus nerve and a strain relief bend of lead to provide slack during movement of the neck was created. The lead connector pin was attached to a generator situated subcutaneously in the left thoracic region. The electrical connections of the whole device were then checked with a system diagnostic performed with the wand and programming computer. During the electro-diagnostic test the pulse generator delivered 1 mA output current at 20 Hz with a pulse width of 500 μsec and measured the impedance. At this moment the heart rate was monitored to highlight a possible bradycardia. The pulse generator was then inserted into the subcutaneous pocket and the two wounds closed in anatomical layers using absorbable suture and paying attention to a good cosmetic result. The generator was switched off for two weeks postoperatively to allow postsurgical edema to resolve.

### Statistical analysis

Subjects’ characteristics were summarized as mean and SD for continuous variables and frequency and percentage for categorical variables. A repeated measures ANOVA was performed on total scores of the HDRS, MADRS and CGI-S scales before (baseline, surgery) and after 3, 6, 9 and 12 months from VNS implant.

## Results

One hundred-fifty-nine subjects were interviewed through telephone in order to assess eligibility criteria for inclusion into the study; of them, 39 were asked to present for a face-to-face clinical interview. Thirty-three patients were excluded because of not having a current Major Depressive Episode (N = 7), not having a chronic (actual episode ≥2 years) or recurrent (history of at least 4 lifetime MDEs) disorder (N = 20), having a medical illness that contraindicated surgery (N = 4), or having a current Substance Use Disorder (N = 2). One additional patient, fulfilling eligibility criteria, first accepted to undergo surgical procedure, and then refused after the screening visit.

**Figure 1:**
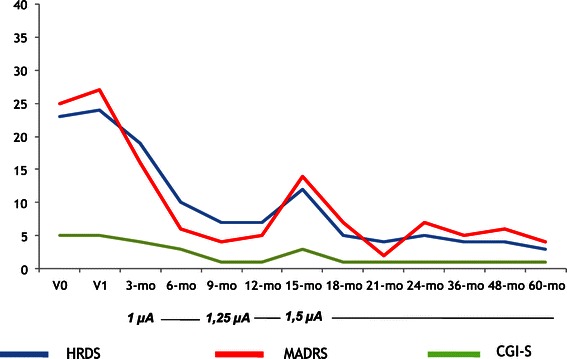
**HDRS, MADRS and CGI-S scores across time for Patient 4 (male, 57 years, Bipolar Disorder type I, comorbid Avoidant and Obsessive-Compulsive Personality Disorders).**

**Figure 2:**
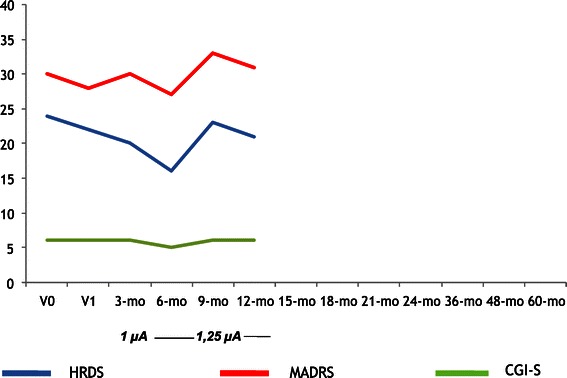
**HDRS, MADRS and CGI-S scores across time for Patient 5 (male, 61 years, Bipolar Disorder type II, no comorbid diagnoses).**

A total of five patients met the inclusion criteria and had the VNS generator implanted. The mean ± SD age of the patients was 56.6 ± 7.3 years (range, 48–66 years). Three patients had a Major Depressive Disorder (60.0%) and the remaining two patients had a Bipolar Disorder (one BD type I and one BD type II). Socio-demographic and clinical data are summarized in Table 1. Concerning the duration of the current MDE, three patients fulfilled DSM-IV criteria for chronic (>2 years) major depression (all three had a diagnosis of unipolar major depression). Pre-implant history of the two BD patients was carefully recorded by means of direct interview, family members’ interview (when available) and medical records review. The first patient (Figure 1) had a history of BD type I with onset at age 26; he had more than 10 lifetime MDEs, with 8 admissions to psychiatric wards and 2 suicide attempts during previous episodes. Despite being on mood stabilizer (valproic acid), during the last two years preceding VNS implant he showed 3 MDEs (not considering the current one) without (hypo) manic episodes, but with intervals between depressive episodes lasting less than 3 months. Treatments with adjunctive lamotrigine or quetiapine did not result in stable remission; adjunctive antidepressants in the current episode did not resolve depressive symptomatology. The second patient (Figure 2) had BD type II, with onset at age 36. He also had more than 10 lifetime MDEs, a history of 1 prior suicide attempt, and had, during the last two years prior to VNS implant, 2 long-lasting MDEs (not considering the current one). He spent, during the last two years prior to surgery, a total of 5 months only without a MDE (although without complete remission), despite being treated with valproic acid, quetiapine, lamotrigine, and four different antidepressants (two of them during the current MDE). No hypomanic episodes were recorded in the last two years.

**Table 1:** **Socio-demographic and clinical characteristics of patients included**

	Sample (N = 5)
Actual age (years) (Mean ± SD)	56.6 ± 7.3
Gender: males, N (%)	2 (40.0)
Educational level (years) (Mean ± SD)	13.4 ± 5.3
Marital status: married, N (%)	2 (40.0)
Currently working, N (%)	4 (80.0)
Age at onset (years) (Mean ± SD)	30.6 ± 4.2
Duration of illness (years) (Mean ± SD)	26.8 ± 5.5
Duration of current MDE (months), (Mean ± SD)	18.4 ± 14.0
Familial history for psychiatric disorders, N (%)	3 (60.0)
Diagnosis, N (%)	
Major depressive disorder	3 (60.0)
Bipolar disorder	2 (40.0)
Number of medications at the time of the implant, (Mean ± SD)	3.0 ± 1.4
Number of antidepressant treatments received before the implant, (Mean ± SD)	8.4 ± 2.7
Number of antidepressant classes received before the implant, (Mean ± SD)	3.6 ± 0.9
Number of MDEs before the implant, (Mean ± SD)	9.4 ± 4.9
Number of hospitalizations before the implant, (Mean ± SD)	7.0 ± 2.4
Number of patients with a history of suicidal attempts before implant, N (%)	3 (60.0)
Prior ECT, N (%)	1 (20.0)

MDE: Major Depressive Episode; ECT: ElectroConvulsive Therapy.

**Figure 3:**
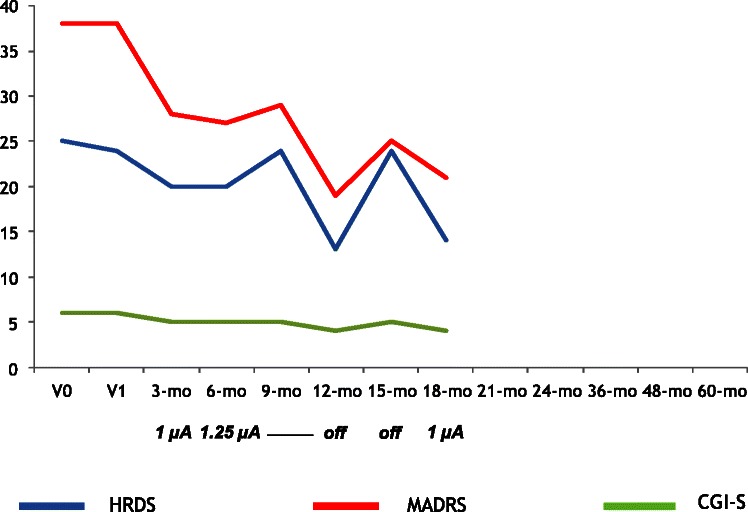
**HDRS, MADRS and CGI-S scores across time for Patient 3 (female, 48 years, Major Depressive Disorder, chronic MDE, no comorbid diagnoses).**

All patients had the VNS generator implanted but for the long-term follow-up, four patients (80.0%) were evaluable at 24 months and three (60.0%) were evaluable at 60 months. Two patients withdrew from the study: one subject (Figure 3), whose depression slightly improved (HDRS-17: 14), withdrew after 18 months because of side effects judged by the patient as intolerable (hoarseness, sore throat and neck pain), and had the VNS generator switched off. The other (Figure 2) withdrew after 12 months because symptoms remained unchanged (HDRS-17: 21); although the clinician in charge of that patient clearly advised him that VNS efficacy sometimes is evident after 12 months of treatment, he was unwilling to attend follow-up assessments and dropped out from the study.

**Figure 4:**
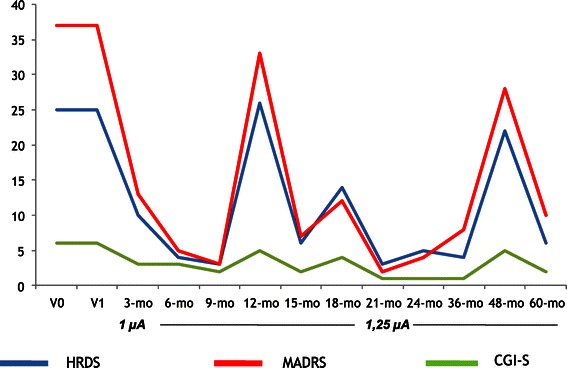
**HDRS, MADRS and CGI-S scores across time for Patient 1 (female, 66 years, Major Depressive Disorder, chronic MDE, no comorbid diagnoses).**

**Figure 5:**
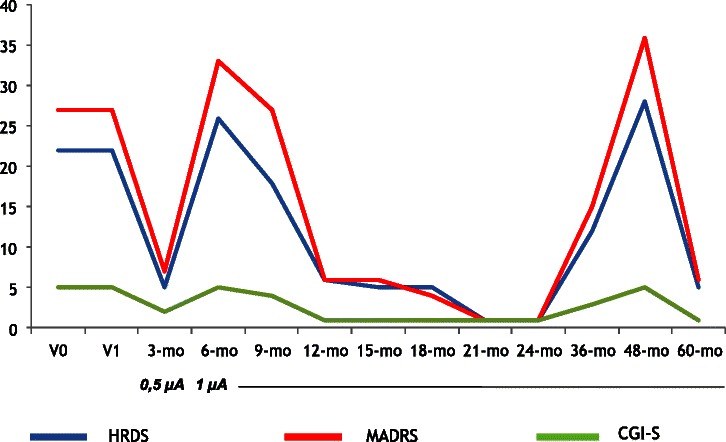
**HDRS, MADRS and CGI-S scores across time for Patient 2 (female, 51 years, Major Depressive Disorder, chronic MDE, no comorbid diagnoses).**

Table 2 shows results of the repeated measures ANOVA on the total scores of the HDRS, MADRS, CGI-S and SF-36 scales (all not statistically significant). Figures 1, 2, 3, 4 and 5 show individual scores for each patient and stimulation parameters, as patients had different outcomes over the 5-year follow-up. According to the HDRS-17, 2 patients were responders (40%) and remitters (40%) after 1 year of VNS treatment (patients 2 and 4). At 2 years, the three patients still on treatment were all responders and remitters. Two of the three patients who were followed-up for 5 years had one depressive recurrence during the 4th year and the third subject showed no recurrences for the whole period.

**Table 2:** **Outcome measures for Intent-to-Treat sample in the first 12 months (N = 5)**

	Baseline (surgery)	3 months	6 months	9 months	12 months	Repeated measures ANOVA	
Variable	Mean (SD)	Mean (SD)	Mean (SD)	Mean (SD)	Mean (SD)	F	p
HDRS-17	23.0 (±2.0)	14.8 (±6.9)	15.2 (±8.6)	15.0 (±9.5)	14.6 (±8.7)	1.120	.382
MADRS	31.4 (±5.6)	18.8 (±9.9)	19.6 (±13.1)	19.2 (±14.5)	18.8 (±13.3)	1.496	.250
CGI-S	5.6 (±0.5)	4.0 (±1.6)	4.2 (±1.1)	3.6 (±2.1)	3.4 (±2.3)	2.429	.090
SF-36 physical summary	42.7 (±7.4)	45.8 (±2.9)	46.3 (±7.1)	43.9 (±2.7)	46.6 (±4.4)	.416	.794
SF-36 mental summary	25.5 (±17.2)	31.7 (±20.4)	31.3 (±15.2)	26.6 (±11.1)	31.7 (±18.4)	.245	.909

HDRS-17: 17-item Hamilton Depression Rating Scale; MADRS: Montgomery Asberg Depression Rating Scale; CGI-S: Clinical Global Impression – Severity of Illness.

Table 3 summarizes the rates of adverse events (AEs) after VNS implant and during the follow-up period. With regard to tolerability, patients reported common side effects traditionally observed in previous clinical studies. The only side effect related to the surgical procedure was neck pain located at the level of the surgical incision, which generally disappeared within 2 weeks from implant. Only in one case the pain persisted up to month 18. Stimulation-related adverse effects experienced by patients were hoarseness, discomfort, sore throat, headache, alteration of voice, and described as mild to moderate. In one patient, hoarseness, sore throat and neck pain were described as severe and intolerable, and the patient asked to have the VNS generator switched off at 1 year; after 5 months we tried again to switch on the generator, but she reported again intolerable side effects and 18 months after the implant she asked to have the generator switched off definitely. Pulse-width was not reduced, although this strategy is commonly used to improve tolerability. No (hypo) manic episodes, a rare but yet documented adverse event potentially occurring with VNS, were observed during the 60 months of stimulation.

**Table 3:** **Side effects of Vagus Nerve Stimulation**

Side effects	Sample (N = 5)
Hoarseness, N (%)	4 (80.0)
Neck pain, N (%)	3 (60.0)
Sore throat, N (%)	2 (40.0)
Headache, N (%)	2 (40.0)
Paresthesia, N (%)	2 (40.0)
Anxiety, N (%)	1 (20.0)
Dysphagia- Dyspepsia, N (%)	1 (20.0)
Others (cough, dyspnoea, chest tightness, stridor, laryngism, reflux, nausea, sweating, earache, snoring), N (%)	3 (60.0)

## Discussion

Our study shows that VNS effectiveness in a clinical practice devoted to the care of individuals with TRD was comparable to the efficacy outcomes reported from prior controlled and uncontrolled clinical trials: the 1-year response and remission rates (based upon the HDRS-17) were 40% and 40% respectively in our case series. Our response rate at 1 year is comparable to the 31.8% response rate (23.2 to 41.8%) after a mean of 20 weeks found in a recent meta-analytical study [38].

To our knowledge, the present study is one of the few prospective ones with a long (5-year for three patients) follow-up of patients who underwent VNS implant for TRD. Findings from our case series indicate a progressive symptom reduction over the first year, although statistical analyses were not significant; moreover, the analysis of individual rating scales scores over time shows that VNS, when effective (not in all patients), is associated with a reduction of the number of depressive recurrences. As already shown by previous reports [21,22,36,39], the present 5-year follow-up study suggests that VNS may prove more useful as a long-term maintenance therapy for chronic depression rather than for acute stabilization of an episode. Preliminary evidence suggests that adjunctive VNS has two principal effects: first, it reduces depressive symptoms in a small but significant proportion of patients who otherwise would be unresponsive to standard treatments, and, second, it reduces exacerbations over the long-term for the vast majority of patients who responded to VNS addition (sustained response) [40]. This long-term effect, however, appears from naturalistic studies or longitudinal pivotal trials without a control or comparison group [22,26,28,36]; long-term observations, being very few in the literature, also small case series like the present one, may be then of interest to clinicians.

Caution has to be used in interpreting our results, as we observed no statistically significant changes in HDRS or MADRS scores (nor in SF-36 scores) in the first 12 months of treatment (see Table 2); moreover, two patients dropped out by 12–18 months. Conclusions about long-term effectiveness (and/or tolerability), being based on the three subjects who remained on treatment, have to be mitigated on the basis of these limitations.

VNS is not a procedure devoid of side effects; one patient from our case series was unable to tolerate hoarseness, sore throat and neck pain, and the VNS generator had to be switched off. Moreover, the majority of patients reported stimulation-related adverse effects, although to a lesser degree. A careful screening of subjects with TRD is then indicated, with particular attention not only to the history of resistant depression (number of previous failed antidepressant trials, compliance, etc.) but also to the history of side effects of previous treatments and to the ability of the patient to tolerate such side effects. Unfortunately, we did not change stimulation settings (e.g. reduced pulse-width) during the treatment phase in order to counterbalance reported adverse effects; we then acknowledge this as a limitation to be kept in mind when interpreting our results concerning side effects of VNS.

One patient refused to attend follow-up assessments after 12 months of treatment, being depressive symptoms unchanged. We acknowledge that a 12-month VNS treatment may be too short to conclude that this procedure is ineffective. Despite several efforts by the psychiatrist in charge of that patient to maintain him in the study, he withdrew the consent and dropped out. Although it is unusual for someone to consent to having a neurosurgical procedure and then refuse to be followed up, he did so and told the psychiatrist that he was willing to try transcranial magnetic stimulation at another University hospital. This case underlines, to our opinion, that a careful screening of subjects with treatment-resistant mood disorders is compulsory before implementing neurosurgical procedures, with a particular attention to motivations to attend complex and expensive procedures such as VNS implantation.

## Conclusions

Our study, even though limited by its small sample size and by its observational nature, shows that VNS may be effective in clinical practice in a small but significant minority of patients. The investigation of potential predictors of VNS response is strongly needed and future studies in larger samples should focus on this matter. Further long-term follow-up studies are also strongly needed in order to confirm the putative stabilizing property of VNS.
